# Designing an Innovative Electrospinning Strategy to Generate PHBV Nanofiber Scaffolds with a Radially Oriented Fibrous Pattern

**DOI:** 10.3390/nano13071150

**Published:** 2023-03-23

**Authors:** Qiuyu Wang, Jianwei Ma, Shaojuan Chen, Shaohua Wu

**Affiliations:** 1 State Key Laboratory of Bio-Fibers and Eco-Textiles, Qingdao University, Qingdao 266071, China; 2 College of Textiles and Clothing, Qingdao University, Qingdao 266071, China

**Keywords:** electrospinning, nanofiber scaffold, fiber alignment, biocompatibility, biomaterials

## Abstract

Electrospinning has contributed substantially to the construction of nanofibrous scaffolds for potential tissue engineering and regenerative medicine applications. However, conventional electrospinning only has the ability to generate and collect nanofiber scaffolds with a randomly oriented fibrous pattern, which lack the necessary cell alignment guidance function. In this study, a novel electrospinning fiber-collecting device was designed and developed by setting a series of small pin-ring-structured collectors on a large plain plate. Specifically, we demonstrated that the pin-ring-structured collectors, which were constructed by inserting a metal pin into the center of a metal ring, could collect the as-electrospun nanofibers with radially oriented structures in an innovative manner. We first investigated the suitable polymeric concentration for electrospinning poly(3-hydroxybutyrate-co-3-hydroxyvalerate) (PHBV), and the optimum electrospinning concentration of PHBV was found to be 12% (*w*/*v*) PHBV dissolved in hexafluoroisopropyl alcohol (HFIP). Then, 12% (*w*/*v*) PHBV solution was electrospun into radially oriented nanofiber scaffolds using our novel electrospinning strategy, and their various performances were further compared with conventionally randomly oriented nanofiber scaffolds that were also produced from 12% (*w*/*v*) PHBV solution. The results showed that the radially oriented PHBV nanofiber scaffolds exhibited obviously enhanced mechanical properties and decreased hydrophobicity compared with the randomly oriented PHBV nanofiber scaffold controls. Importantly, the biological properties of radially oriented PHBV nanofiber scaffolds were also demonstrated to be enhanced, compared with randomly oriented PHBV nanofiber scaffolds, by effectively inducing cell alignment and significantly promoting cell proliferation. In sum, the present study indicates that our as-prepared nanofiber scaffolds with a radially oriented pattern are of great interest for advanced applications, such as wound dressings and tissue-engineered scaffolds.

## 1. Introduction

The vast majority of tissues and organs in the human body lack self-regenerating or -repairing capacities after physical injuries or damage from disease, which make the damage or injuries of tissues and organs irreversible [1]. Although tissue and organ transplant has contributed greatly to saving patients’ lives, it is difficult for this medical strategy to cope with rapidly growing demand and a large paucity of donors. It was reported that over 100,000 people were on the waiting list of the national register in the USA alone [2]. In the last several decades, tissue engineering that designs and develops biological substitutes to restore, maintain, or improve tissue function has been recognized as a promising alternative to previous allogeneic organ transplantation in clinics [3]. The construction of advanced scaffolds that can replicate the native extracellular matrix (ECM), to a large extent, has aroused intense interest in the fields of tissue engineering and regenerative medicine, because ECM plays significant roles in maintaining cell survival and tissue function in natural tissues and organs [4,5].

Recently, electrospinning has been widely explored to produce fibers with diameters in a range from several nanometers to several hundred nanometers, which is of great interest for fabricating biomaterial scaffolds used in tissue engineering because the electrospun nanofibers have the most similar structure and performance to collagen fibrils in the native tissue ECM [6,7]. Previous studies have demonstrated that the fibers generated by electrospinning could dramatically promote cell adhesion, proliferation, and even directed differentiation, thus resulting in obviously enhanced tissue-regenerative outcomes in animal studies when compared with the fibers fabricated from traditional spinning methods such as melt spinning [8,9]. According to the statistics, more than one hundred different types of polymers from natural or synthetic sources have been successfully electrospun into nanofibers, and researchers can precisely modulate the biocompatibility, biodegradation, and physical and chemical properties of electrospinning-based tissue scaffolds by selecting different spinning compositions [10,11]. Moreover, electrospinning can also be treated as an effective carrier, and various drugs, bioactive factors, and even living cells have been successfully loaded into or onto electrospun nanofibers, in order to improve the biological functions of as-prepared nanofiber scaffolds [12,13].

PHBV, a copolymer consisting of hydroxyvalerate and hydroxybutyrate with different proportions, can be synthesized through the fermentation process of Gram-negative bacteria [14]. Recent studies have found that PHBV exhibits excellent biocompatibility and biodegradability, and its degradation product, D-3-hydroxybutyric acid, is an important component in human blood [15,16,17]. Importantly, compared with petroleum-based biopolymers, PHBV is a suitable candidate for renewable resources for a wide range of biomedical applications due to its outstanding environmental friendliness [18,19].

**Figure 1:**
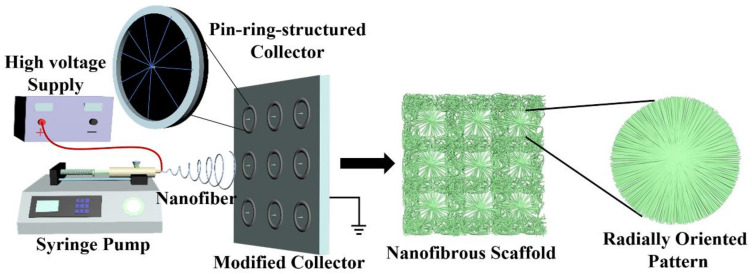
Schematic of our innovative electrospinning device that employs a series of pin-ring-structured collectors as an electrospun nanofiber-collecting device to generate a radially oriented nanofiber pattern.

As electrospun nanofibers are usually collected with randomly aligned fiber structures in a chaotic manner, their cell recruitment and guidance capabilities can still be greatly improved [20]. To address this issue, a specially designed fiber-collecting device was designed and implemented to harvest electrospun nanofibers in the form of radially oriented structures, as shown in Figure 1. PHBV, a popular biomaterial, was selected as a model polymer to verify the feasibility of our as-designed electrospinning strategy. As a preliminary study, the spinning concentrations of PHBV were first explored. Then, PHBV with predetermined concentration was electrospun into two different patterns, i.e., randomly oriented pattern and radially oriented pattern. We systematically compared the structure and physical, mechanical, and biological performances of these two different fiber patterns. This paper aims to provide guidance for the innovative development of electrospinning-based scaffolds with a radially oriented nanofiber pattern.

## 2. Materials and Experimental Methods

### 2.1. Materials

PHBV (Mw = 30,000, 3% HV) was obtained from Nanjing Hesu Times New Material Technology Company (Nanjing, China). HFIP (purity ≥99.8%) was purchased from Shanghai Aladdin Reagent (Shanghai, China). All the chemicals were used as received, without further purification.

### 2.2. Design of a Novel Electrospinning Strategy

Series of small metal collectors with a pin-ring structure were constructed by inserting one copper pin into the center of one copper ring, which were then fixed onto a plain plate to generate a novel electrospinning fiber-collecting apparatus, as shown in Figure 1. Together with a syringe pump (Longer Constant Flow Pump Co. LTD, Hebei, China), a blunt-tip needle (18 G) connected to a medical syringe (10 mL), and a high-voltage supply (Gamma, Westmont, IL, USA), one whole electrospinning device was assembled by our own lab.

### 2.3. Optimization of Spinning Concentration of PHBV

HFIP was chosen as the solvent for the preparation of PHBV solution based on previous work [19,21]. PHBV was dissolved into HFIP by using a magnetic mixer, in order to obtain three spinning solutions with different concentrations, i.e., 9%, 12%, and 15% (*w*/*v*). The as-prepared PHBV solutions with different concentrations were electrospun into nanofiber scaffolds with a randomly orientated pattern by using a conventional plain plate as a fiber collector. The same electrospinning parameters were employed. Specifically, the spinning voltage, solution feeding rate, and collection distance were fixed at 15 kV, 0.8 mL/h, and 18 cm, respectively. Following this, the obtained PHBV nanofiber scaffolds were vacuum dried for 72 h.

### 2.4. Preparation of PHBV Nanofiber Scaffolds with a Radially Oriented Pattern

We selected a 12% (*w*/*v*) PHBV solution for the spinnability test of our designed electrospinning strategy, described in the Section 2.2. Using our electrospinning fiber-collecting device by setting a series of small pin-ring-structured collectors on the large plain plate as a fiber collector, the spinning voltage, solution feeding rate, and collection distance were fixed at 15 kV, 0.8 mL/h, and 18 cm, respectively. The fibers deposited on both the area of the small pin-ring-structured collector and other areas of the large plain plate were harvested, vacuum dried, observed, and characterized.

### 2.5. Morphological Observation

A scanning electron microscope (Regulus 8100, Hitachi, Japan) was employed to observe the morphology of each as-fabricated PHBV nanofiber scaffold. The samples were cut into small pieces and attached to the sample table with conductive adhesive and further conducted with a gold spraying process for 80 s. For each sample, at least three SEM images were selected, and 100 fibers were chosen in a random way to measure the fiber diameters.

### 2.6. Fourier-Transform Infrared (FTIR) Analysis

The chemical groups of PHBV nanofiber scaffolds with different fiber patterns were characterized using a Bruker TENSOR27 FTIR machine (Borken, Germany). The scanning range was 4000~500 cm^–1^, and the scanning resolution was set to 4 cm^–1^.

### 2.7. Mechanical Characterization

**Figure 2:**
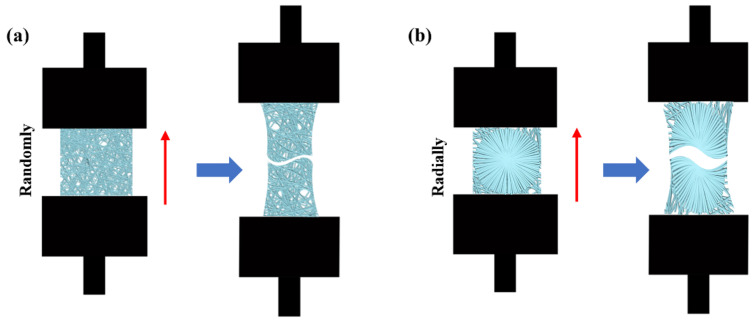
Schematic of tensile test: (**a**) randomly oriented nanofiber scaffolds; (**b**) radially oriented nanofiber scaffolds.

The mechanical properties of PHBV nanofiber scaffolds with two different patterns were measured by using an INSTRON 5965 tensile testing machine (USA). A tensile schematic of the two different patterns is shown in Figure 2. The two different samples were randomly cut into a series of strips, 3 cm long and 1 cm wide. During the test, the clamping distance was 1 cm, which was exactly the radial circle diameter of the radially oriented pattern. Each sample was tested at a tensile rate of 1 mm/min until failure occurred. The initial modulus, ultimate strength, and strain to failure were calculated and characterized.

### 2.8. Water Contact Angle Analysis

The surface hydrophilicity of as-prepared PHBV scaffolds with different nanofiber patterns was determined with a fully automatic contact measuring instrument (XG-CAMD3, Shanghai Xuanjun Instrument, Shanghai, China). A droplet of deionized water (2 mL) was applied on the surface of the PHBV sample, and the change in water contact angle was recorded over time until equilibrium status was reached.

### 2.9. Cell Characterization and Material Biocompatibility Test

Human-adipose-derived mesenchymal stem cells (HAMSCs) were purchased from the Chinese Academy of Sciences (Shanghai, China), and they were utilized as model cells to characterize and compare the biocompatibility of PHBV scaffolds with randomly or radially oriented nanofiber patterns. The cells were cultured with the DMEM/F12 medium (Gibco, Shanghai, China) containing 10% fetal bovine serum (FBS, Gibco, Shanghai, China) and 1% penicillin/streptomycin (P/S, Gibco, Shanghai, China). The different PHBV samples were punched into circle-like structures with a diameter of 10 mm and sterilized in 75% ethanol overnight. After multiple washes using sterilized phosphate buffer saline (PBS) solution, the samples were immersed in the cell culture medium overnight. Then, the HAMSCs were seeded on the as-sterilized samples with a density of 1 × 10^4^ cells, and they were cultured in a 5% CO_2_-contained incubator at 37 °C. The medium was changed every two days until the predetermined time point was reached.

IFluorTM 488 phalloidin and DAPI staining were used to observe cell morphology and nuclei. In detail, the HAMSC-seeded PHBV scaffolds were fixed with 4% paraformaldehyde solution at 4 °C for 4 h after 7 days of culture. The as-fixed cell scaffold samples were immersed in 0.2% Triton-X100 at room temperature for 10 min and then transferred to 1% BSA for 24 h at 4 °C. Then, IFluorTM 488 phalloidin dye (1:200, Yeasen Biotechnology, Shanghai, China) was utilized to stain the samples at room temperature in the dark for 2 h, and DAPI (1:1000, Thermo Scientific, Boston, MA, USA) was further employed to stain the nuclei at room temperature in the dark for 30 min. The stained samples were then observed with a confocal laser scanning microscope (CLSM, Zeiss 900, Carl Zeiss, Baden-wurttemberg, Germany).

The viability and proliferation of HAMSCs when seeded and cultured on the PHBV scaffolds with different oriented patterns were determined by using a MTT assay (Sigma, Beijing, China). The HAMSC-seeded PHBV samples were harvested after 1, 3, and 7 days of culture and transferred into 24-well cell culture plates in which each well contained 100 μL 5 mg/mL MTT solution and 1 mL medium. After 4 h of incubation, the formazan crystals were totally formed in the living cells, and 500 μL/well dimethyl sulfoxide (DMSO) was added to completely dissolve the formazan crystals. Finally, 100 μL formazan/DMSO solution was taken from each well, and a microplate reader (Infinite M Nano, Tecan, Mannedorf, Switzerland) was utilized to read the OD value at a wavelength of 490 nm.

### 2.10. Statistical Analysis

The experimental data in this study ensured that each group contained at least three duplicate samples, and all quantitative data were presented as mean ± standard deviation. Statistical analysis was conducted using an unpaired *t*-test, and *p* < 0.05 was recognized as a significant difference.

## 3. Results and Discussion

The application of nanotechnology such as electrospinning to design and construct biomaterial scaffolds is a promising trend in the fields of tissue engineering and regenerative medicine [22]. Currently, the conventional electrospinning device commonly employs a metal plain plate or low-speed roller to collect electrospun nanofibers, and in this context, the electrospun nanofibers are harvested in a totally chaotic and randomly deposited manner [23,24,25]. It is well known that the morphology and structure of tissue-engineered scaffolds play important roles in physical and biological performance [26,27]. To improve the functions of electrospun nanofibers, some previous studies have developed innovative strategies to control the deposition and orientation of electrospinning nanofibers [28,29]. Among them, modifying the electrospinning fiber-collecting device has attracted much attention. For instance, a high-speed metal roller and two oppositely placed metal electrodes with a constant gap, etc., have been developed as advanced electrospun fiber collectors to control the alignment of electrospinning nanofibers to fabricate electrospun scaffolds with uniaxially oriented nanofibrous patterns [30,31]. In this study, one new-type electrode with a pin-ring-like structure was designed and implemented to collect electrospun nanofibers in the form of a radially oriented structure. In detail, one metal pin was inserted into the center of one metal ring, and a series of small pin-ring-like collectors was then set on one large plain plate in an array architecture, which was utilized as a modified electrospun fiber collector. A detailed schematic is shown in Figure 1, and except for the fiber collector, the other parts are similar to the conventional electrospinning device. The pin-ring-structured collector was expected to change the electric field distribution in the gap formed by a centered pin and a peripheral ring, and to further control the radially oriented deposition of electrospun nanofibers.

### 3.1. Morphological Observation of Electrospun PHBV Scaffolds with a Radially Oriented Pattern

**Figure 3:**
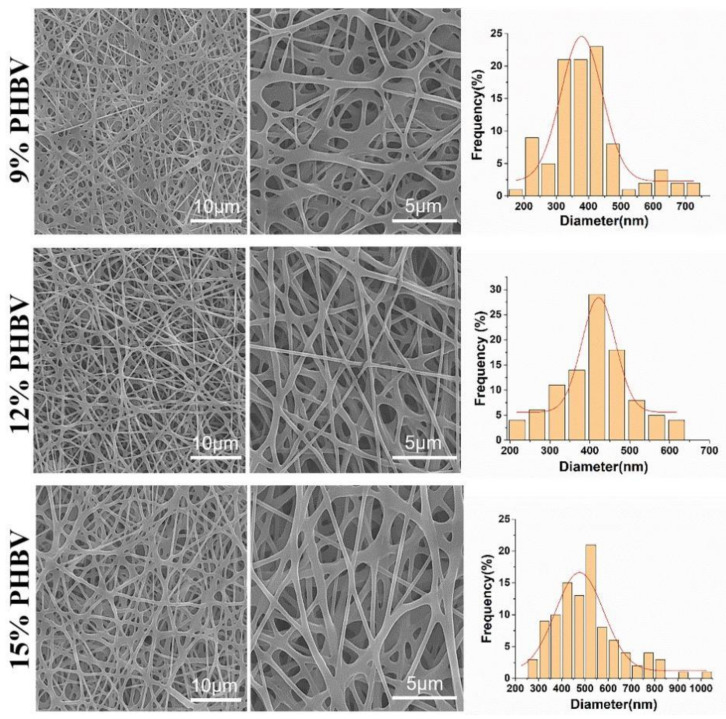
SEM images and corresponding diameter distribution analysis of PHBV nanofiber scaffolds produced from three different polymeric concentrations, i.e., 9%, 12%, and 15% (*w*/*v*).

PHBV was chosen to demonstrate the feasibility of our as-designed electrospun fiber collector. The spinnable concentration of PHBV was first explored by using a conventional electrospinning device that employed a common metal plain plate as a collector. The SEM images in Figure 3 show the morphology of electrospun PHBV nanofiber scaffolds produced from three different polymeric concentrations, i.e., 9%, 12%, and 15% (*w*/*v*). It was found that no bead-like structure was found in all three PHBV samples, and the fibers in the 12% PHBV sample exhibited obviously higher evenness compared with both the 9% and 15% PHBV samples. The results of the fiber diameter analysis further confirmed that more uniform fibers were obtained in the electrospun PHBV scaffolds produced from the polymeric concentration of 12%. Moreover, the mean fiber diameters of 9%, 12%, and 15% PHBV scaffolds were calculated to be 388.3 ± 115.9 nm, 402.8 ± 107.9 nm, and 510.9 ± 145.4 nm, respectively, indicating that the nanofiber diameter presented an increasing trend with an increase in PHBV concentration. Taken together, 12% PHBV dissolved in HFIP solvent was chosen for our following studies.

**Figure 4:**
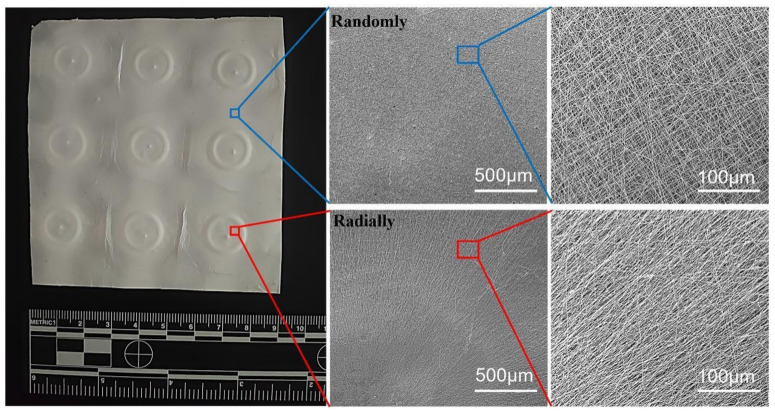
Photograph and SEM images of as-prepared PHBV nanofiber scaffolds with both randomly and radially oriented patterns.

Our novel electrospinning device, as shown in Figure 1, was utilized to electrospin 12% PHBV/HFIP solution into nanofibers, and the as-collected nanofibers are presented in Figure 4. It clearly shows that the nanofibers deposited in the small pin-ring-constructed collectors presented obvious radially oriented fiber morphology. In other words, all fibers showed interesting alignment from all sides to the circle center, which demonstrated our hypothesis that our novel pin-ring-constructed collectors could control the deposition of electrospun PHBV nanofibers and collect the nanofibers in a radially oriented manner. In comparison, the nanofibers deposited on other areas of the large plain plate were found to exhibit common randomly oriented structures, similar to the most conventional electrospun nanofiber structures.

### 3.2. FTIR Analysis of Electrospun PHBV Nanofiber Scaffolds with Both Randomly and Radially Oriented Patterns

**Figure 5:**
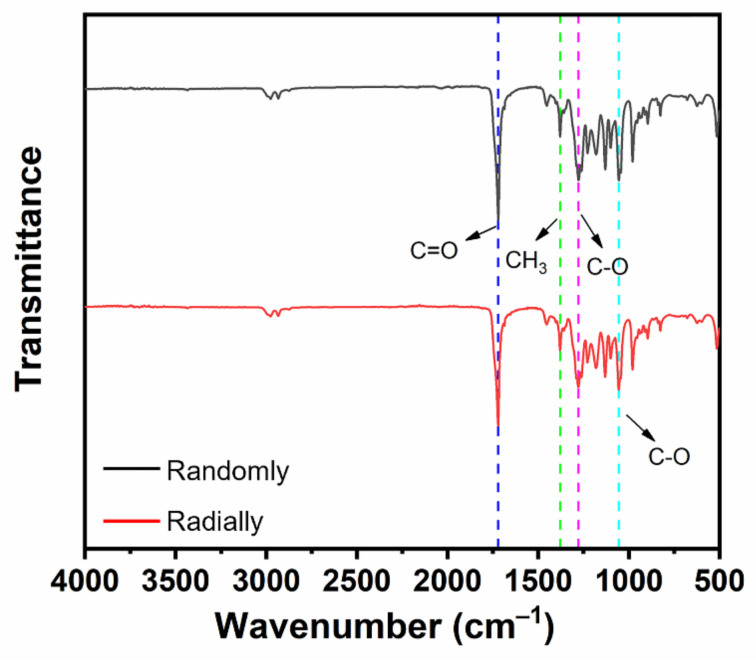
FTIR spectra of as-prepared PHBV nanofiber scaffolds with both randomly and radially oriented patterns.

The chemical groups of electrospun PHBV nanofiber scaffolds with both randomly and radially oriented patterns were determined with FTIR characterization. As shown in Figure 5, both samples exhibited similar curves. In detail, the stretching vibration peak observed at 1721 cm^–1^ belonged to the ester carbonyl (C=O) of the PHBV polymer, and the peak observed at 1379 cm^–1^ was attributed to the symmetrical wagging of the aliphatic methyl (CH_3_) of the PHBV polymer [32]. Moreover, the symmetric stretching vibration peaks of C-O in PHBV were also observed at 1278 cm^–1^ and 1055 cm^–1^ [33]. It was found that all typical peaks of PHBV were detected in both randomly and radially oriented electrospun PHBV scaffolds, and no obvious peak shifting phenomena were observed, indicating that the fiber-oriented pattern did not affect and change the chemical groups of as-prepared PHBV nanofiber scaffolds.

### 3.3. Mechanical Properties of Electrospun PHBV Nanofiber Scaffolds with Both Randomly and Radially Oriented Patterns

**Figure 6:**
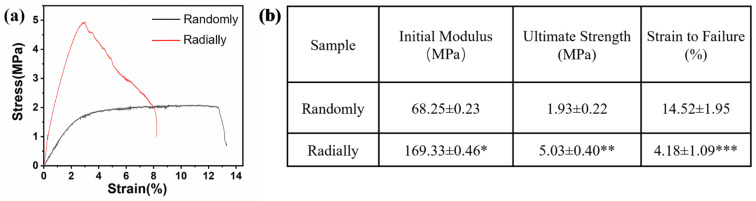
Mechanical properties of as-prepared PHBV nanofiber scaffolds with both randomly and radially oriented patterns: (**a**) representative tensile stress–strain curves; (**b**) analysis of some specific mechanical parameters (n = 5; * *p* < 0.05, ** *p* < 0.01, *** *p* < 0.001).

The results of the mechanical test showed that the electrospun radially oriented PHBV nanofiber scaffolds had completely different tensile curves to the electrospun randomly oriented PHBV nanofiber scaffolds (Figure 6a). Specifically, the randomly oriented PHBV nanofiber scaffolds exhibited a linear elastic region and a subsequent yield platform region until breaking occurred. However, no obvious yield platform region was found for the electrospun radially oriented PHBV nanofiber scaffolds. Instead, some fibers began to break in a gradual way after the primary linear elastic region. This phenomenon was attributed to the radially oriented fiber pattern. Combined with the findings of previous studies [34,35], we demonstrated that fiber alignment had a large effect on the mechanical behavior of as-prepared scaffolds. Figure 6b further compares the key mechanical parameters of the two different scaffolds. The radially oriented nanofiber pattern showed obviously enhanced initial modulus and ultimate strength compared with those of the randomly oriented nanofiber pattern (169.33 ± 0.46 MPa vs. 68.25 ± 0.23 MPa and 5.03 ± 0.40 MPa vs. 1.93 ± 0.22 MPa, respectively). Moreover, the strain to failure of the radially aligned nanofiber pattern was much lower than the randomly oriented nanofiber pattern (4.18 ± 1.09% vs. 14.52 ± 1.95%). Our results indicated that fiber alignment could improve the mechanical properties of as-electrospun PHBV nanofiber scaffolds to some extent, which is beneficial for improving structural stability when serving as tissue-engineered scaffolds.

### 3.4. Hydrophilicity Analysis of Electrospun PHBV Nanofiber Scaffolds with Both Randomly and Radially Oriented Patterns

**Figure 7:**
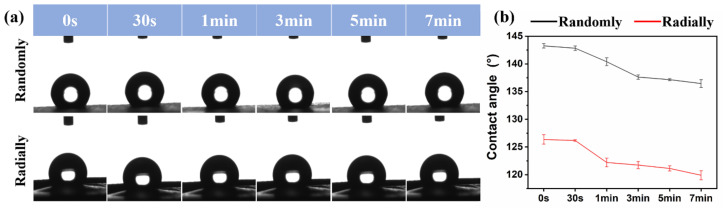
Water contact angles of as-prepared PHBV nanofiber scaffolds with both randomly and radially oriented patterns: (**a**) actual photographs; (**b**) statistical analysis.

The surface hydrophilicity of as-prepared PHBV randomly and radially aligned nanofiber scaffolds was investigated via the water contact angle test, and the relevant results are presented in Figure 7. The initial water contact angles of PHBV randomly and radially aligned nanofiber scaffolds were ~143° and ~126°, respectively. One previous study showed that the water contact angle of electrospun PHBV randomly aligned nanofiber scaffolds was ~141° [36], which is very similar to our result. When equilibrium was progressively reached, the water contact angle was ~137° for PHBV nanofiber scaffolds with the randomly oriented pattern and ~120° for PHBV nanofiber scaffolds with the radially oriented pattern. These results indicated that, although the electrospun PHBV nanofiber scaffolds were hydrophobic materials, the PHBV randomly aligned nanofiber scaffolds exhibited obviously decreased hydrophobicity compared with the PHBV radially aligned nanofiber scaffolds. As potential biomaterial scaffolds for tissue-engineering applications, their surface hydrophilicity is of significant importance, because many previous studies demonstrated that cell adhesion, proliferation, and migration were largely influenced by the surface hydrophilicity of tissue-engineered scaffolds [37,38,39]. Some existing studies demonstrated that a relatively hydrophilic surface was beneficial for the initial attachment and subsequent migration and proliferation [40]. In addition, scaffolds with a hydrophilic surface could be suitable for melting with host tissues after in vivo implantation [41]. Many different factors, mainly including material component and morphology, can affect the surface hydrophilicity of scaffolds [38,42]. Our study demonstrated that fiber orientation had large effects on the electrospun PHBV scaffolds, and our radially aligned nanofiber scaffolds exhibited obviously increased hydrophilicity compared with the conventional randomly aligned nanofiber scaffolds. Therefore, the advanced radially aligned nanofiber pattern seems to be a better choice of biomaterial scaffold.

### 3.5. Biocompatibility of Electrospun PHBV Nanofiber scaffolds with Both Randomly and Radially Oriented Patterns

**Figure 8:**
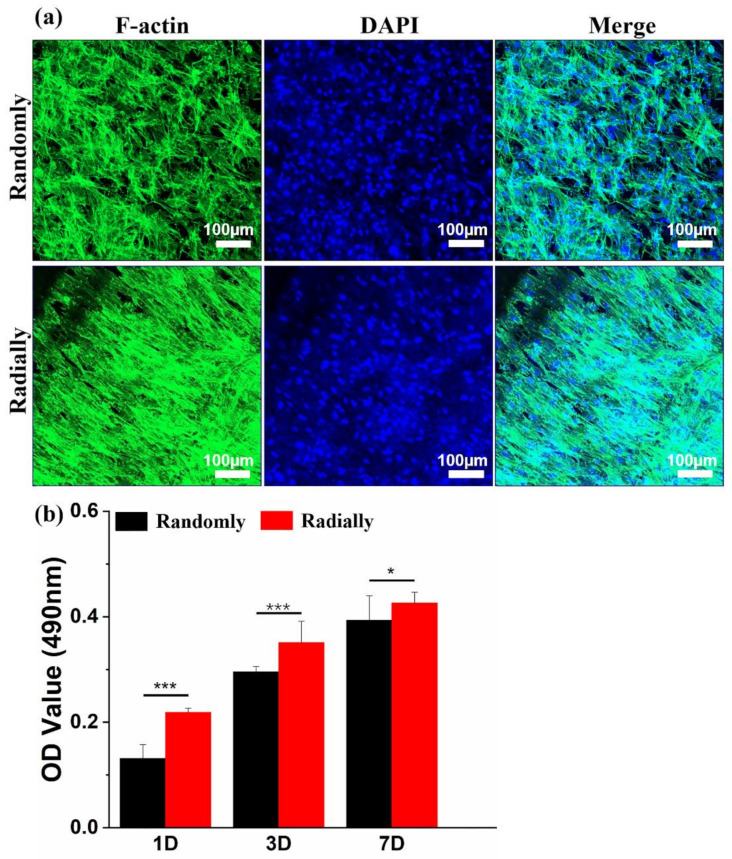
(**a**) Fluorescent images of F-actin (green) and nuclei (blue) staining on day 7 after HAMSCs were seeded and cultured on PHBV nanofiber scaffolds with both randomly and radially oriented patterns. (**b**) MTT results after HAMSCs were seeded and cultured on two different samples over 7 days (n = 5; * *p* < 0.05, *** *p* < 0.001).

HAMSCs were chosen as a model cell type to determine and compare the cytocompatibility difference between PHBV randomly oriented nanofiber scaffolds and radially oriented nanofiber scaffolds, because HAMSCs are readily available and widely utilized in various tissue-engineering applications, such as bone, tendon, cartilage, neural, and cardiovascular regeneration [43]. As shown in Figure 8a, the cytoskeleton and nuclei of HAMSCs were visualized by staining with green and blue, respectively, when HAMSCs were seeded and cultured on the two different PHBV scaffolds for 7 days. It was clearly found that cells cultured on the randomly oriented fiber pattern presented totally ruleless and chaotic cell elongation and orientation. In comparison, cells spread along the fiber alignment direction and exhibited regular arrangement when cultured on the radially aligned fibrous pattern. This is highly consistent with some previous studies that cells can respond to scaffold morphology and further reshape themselves along the arrangement direction of electrospun nanofibers [9]. A classical MTT assay was further employed to investigate the viability and proliferation of HAMSCs when seeded and cultured on the two electrospun PHBV nanofiber scaffolds with randomly or radially oriented patterns. The results from Figure 8b showed that cells on radially oriented nanofiber scaffolds exhibited dramatically higher OD values than those on randomly oriented nanofiber scaffolds after 1, 3, and 7 days of culture, which indicated that the radially oriented nanofiber pattern was more appropriate for cell adhesion and proliferation.

In sum, our study demonstrated the feasibility of constructing PHBV nanofiber scaffolds with an innovative radially oriented pattern, and we further confirmed significantly improved cell regulation properties, such as inducing cell alignment and promoting cell attachment and proliferation of the radially oriented pattern, in comparison with the conventionally randomly oriented pattern. Previous review work has already summarized the different strategies of fabricating electrospun uniaxially aligned nanofiber scaffolds and demonstrated the merits of the uniaxially oriented patterns compared with the randomly oriented pattern for tissue-engineering applications [44]. In this study, our radially aligned nanofiber scaffolds are of great interest as potential candidates for tissue-engineering applications, especially for those tissues that possess radially arranged ECM fibril structures, such as meniscus, heart valve leaflets, etc. [45,46]. They may serve as one scaffold layer for the design and development of these complicated tissue scaffolds. In addition, radially aligned nanofiber scaffolds may be good candidates for advanced wound-dressing materials. Many previous studies have demonstrated that the uniaxially oriented pattern could significantly increase wound closure by inducing cell migration along the fiber alignment from the sides to the center [34,47]. In comparison, our radially oriented pattern is expected to speed up the healing process of various wounds by guiding rapid cell migration along the “expressway” offered by radially aligned fibers from the wound periphery into the wound center. Further in vitro cell characterization and in vivo animal model verification should be performed in the future.

## 4. Conclusions

A novel electrospinning strategy that employed a series of pin-ring-structured collectors as an electrospun nanofiber-collecting device was designed and implemented, and PHBV was successfully electrospun into nanofiber scaffolds constructed with radially oriented nanofibers. The radially oriented nanofiber pattern was found to dramatically increase the hydrophilicity and mechanical properties of electrospun nanofibers compared with the conventionally randomly oriented nanofiber pattern. Moreover, the radially oriented nanofiber pattern was also demonstrated to effectively induce the alignment and dramatically promote the adhesion and proliferation of HAMSCs compared with the randomly oriented nanofiber control. Our study aims to provide meaningful reference for the advanced development of electrospun nanofiber scaffolds with a radially aligned fiber pattern, which shows huge potential for the application of novel wound dressings and various tissue-engineered scaffolds.

## Data Availability

Data are available from the corresponding author upon request.
